# Hepatectomy as a Salvage procedure for blunt abdominal trauma: A case report^☆^

**DOI:** 10.1016/j.amsu.2022.103356

**Published:** 2022-02-06

**Authors:** Eduardo C. Ayuste, Siegfredo R. Paloyo, Allan Dante M. Concejero, Joel U. Macalino, Orlando O. Ocampo

**Affiliations:** a University of the Philippines-Philippine General Hospital Department of Surgery, Philippines; b College of Medicine, University of the Philippines-Manila, Philippines

**Keywords:** Case report, Blunt abdominal injury, Non-operative management, Hepatectomy

## Abstract

The liver is the organ most commonly injured in blunt abdominal trauma. Significant changes in the management of liver trauma have occurred over the last four decades with non-operative management being the first-line of treatment. Although hepatic resection for trauma is an accepted and established option for definitive treatment, it is rarely performed because of the associated morbidity and mortality, at least historically. Herein we describe a case of a 24-year old male who had blunt abdominal injury for which a right hepatectomy was eventually performed after an initial attempt at damage control surgery. We would like to highlight that early decision by a dedicated team of surgeons coupled with the necessary support from ancillary services as well as coordination between trauma surgeons led to a successful outcome in this case. This case presents an opportunity to revisit the role of hepatic resection in the management of complex liver injuries.

## Highlights

• Liver is a frequently injured organ in blunt or penetrating injury.
• Non-operative management can be successful in majority of cases.
• Nonanatomic or anatomic hepatic resection can be a treatment option in select cases.
• Timely decision is paramount in the success of operative management for blunt trauma.

## 1 Introduction

The liver is a frequently injured organ in blunt as well as penetrating trauma for which a non-operative management is generally initially attempted and most often would prove to be a definitive plan of action. Hepatic resection for trauma is rare and has historically been associated with a high mortality and has thus consigned this procedure, for the most part, into the recycling bin of surgical techniques. Herein we describe a case managed at a high-volume academic trauma center for which a selective operative approach was undertaken exemplifying that hepatic resection may be reserved for those instances where bleeding cannot be arrested by packing or damage control surgery. The case presents an opportunity to revisit the role of hepatic resection in the management of complex liver injuries. This report has been written in line with the recent SCARE criteria for case reports [1].

## 2 Case

**Fig. 1:**
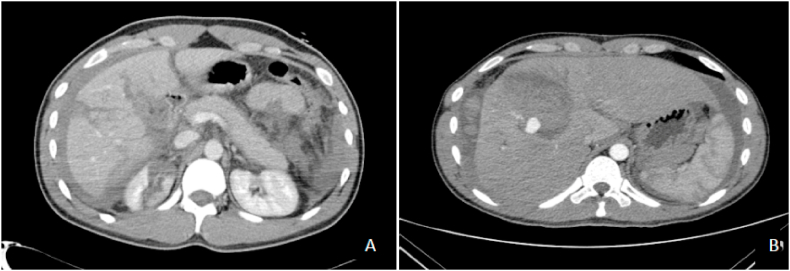
CT scan findings showing hepatic capsular and parenchymal laceration affecting segments V, VI and VIII (A) with subcapsular hematoma (B) formation at posterior aspect of right liver lobe.

**Fig. 2:**
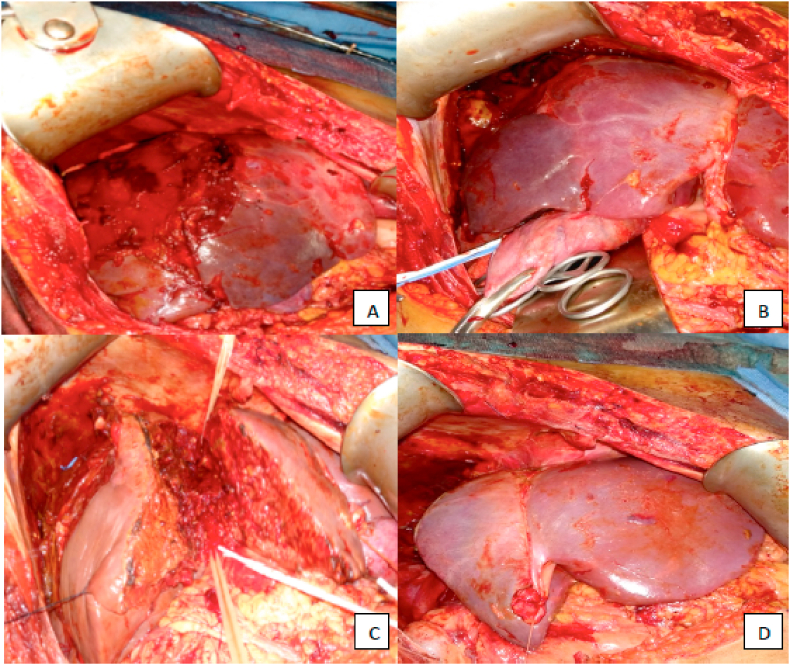
Intraoperative findings showing the extent of hepatic injury on the right hepatic lobe (A) and demarcation (B). Hepatic resection using finger-fracture technique (C). Remaining hepatic lobe after resection (D).

A 24-year-old male with no known comorbidities figured in a motorcycle crash in an island resort 1400 km away. He was initially managed at a local hospital wherein they performed damage control surgery (perihepatic packing, blood transfusion, ICU management) before being airlifted to our institution. Initial FAST (Focused Assessment with Sonography in Trauma) was positive for fluid at the hepatorenal space while an abdominal CT scan done prior revealed liver parenchymal laceration in segments V, VI and VIII (AAST (American Association for the Surgery of Trauma) Grade IV) and right renal laceration (AAST Grade III) with hemoperitoneum (Fig. 1A and B). He was received 2 weeks post-injury with a GCS of 15, BP 100/70 and HR of 70 and an Injury Severity Scale (ISS) of 26. The plan was to continue ICU management however, on the 2nd hospital day, patient developed episodes of hypotension and generalized abdominal tenderness with guarding necessitating re-laparotomy and performing a right hepatectomy (Fig. 2A–D). Intraoperatively, there was 1.5L of hemoperitoneum with note of 12 cm deep stellate laceration of the liver involving the aforementioned segments with intact biliary tree. Patient recovered well, and was discharged after 12 days with no complications. At 1 year of follow-up, the patient is doing well and back to work as an environmental photographer.

## 3 Discussion

The management of blunt liver injuries have evolved over the last four decades. Early experience with hepatic resection for trauma yielded high mortality rates (>50%) which led to the procedure being deprecated. The reemergence of perihepatic packing and influx of modern diagnostic and therapeutic tools ushered in a new option of non-operative management (NOM) which can be successfully applied in >80% of cases [2]. The improvements in CT imaging, utility of interventional radiology such as angioembolization and endoscopic retrograde cholangiopancreatography (ERCP) with stenting were contributing factors in decreasing mortality with such treatment option. However, some would argue that this strong tendency towards non-operative management, particularly for high-grade liver injuries (AAST Grade IV-V) lead to increasing morbidity [3].

Recent guidelines recommend NOM as the treatment of choice for all hemodynamically stable liver trauma patients regardless of AAST grade in the absence of other internal injuries requiring surgery [3]. In patients considered transient responders with AAST Grade III**–**V liver injuries, NOM should be considered only in selected settings provided there are available trained surgeons, operating room, intensive care unit (ICU), access to angiography, blood products, and in locations where a system exists to quickly transfer such patients to higher level of care facilities. Our patient was airlifted from an island as the previous institution had limited angiogram and ICU facility, lacked a complement of specialty surgeons as well as ancillary services. Coordination between the two hospitals and airlift availability led also to the delay in transport which, when taken all together, may have contributed to suboptimal surgical management. Within 48 hours upon arrival at our institution, while in the ICU, hemodynamic instability led to the shift of approach to operative management. An anatomic resection (right hepatectomy) was performed by a team of trauma, hepatobiliary and liver transplant surgeons. There were significant necrotic tissues in the involved liver segments and most bleeding came from its parenchyma.

Over the years, with the improvement of our understanding on liver anatomy and physiology, advances in critical care and the advent of liver transplant teams, hepatic resections have become safe in many centers worldwide. Although operative mortalities for high-grade liver injuries remain high in the literature, several reports have concluded otherwise especially in centers who have more experienced liver and transplant surgeons. Strong et al. reported mortality rates after hepatic resection of 11.1% with liver-related morbidity of 19% and emphasized the use of simple methods to successfully treat injuries while resection, if needed, be performed by a dedicated liver team [4]. Tsugawa et al. reported a larger series of 100 patients with mortality rates of 24% [5]. This study highlighted the differences in the mechanism, grade of injury, and complications between the elderly and younger population and noted that the high survival rate among the elderly supports resection as a safe option for this subpopulation. More recently, Polanco et al. reported 56 patients with hepatic trauma AAST Grade III to V, predominantly blunt injuries, with mortality rates of 9% and concluded that resection can be applied as an initial or delayed plan of management [6]. The advantages of liver resection are that it will most effectively address hemorrhage, remove devitalized tissues, and lessen morbidity associated with bile leak particularly for complex liver injuries.

Several risk factors have been associated with non-survival with the grade of liver injury being a strong predictor of mortality. Doklestic et al. studied 121 hepatic trauma patients with AAST Grade III-IV injuries and showed that non-survivors had higher grade of injury, higher liver enzyme level, significant hypotension, higher Injury Severity Score (ISS) score and lower Glasgow Coma Scale (GCS) on arrival [7]. They also noted that more blood transfusions were given within the first 24 hours and concluded that prolonged bleeding and amount of transfusions were also statistically significant predictors of mortality in severe hepatic trauma. Uribe et al. further demonstrated that a lower Revised Trauma Score (RTS) and the presence of associated intra-abdominal injuries were also independent risk factors of outcome [8].

The general approach to hepatic injury at our center is to attempt non-operative management for blunt injuries regardless of grade of injury. We utilize FAST to evaluate presence of intraabdominal fluid and proceed with diagnostic peritoneal lavage (DPL) assessing for hollow viscus injury (i.e. presence of bile, succus, food particles, urine and bacteria). Hemodynamic instability, presence of peritoneal signs or a positive DPL would prompt a shift in management to operative intervention. Recently, we have expanded our armamentarium and involved interventional radiologists in selected cases of complex injuries. If the patient requires laparotomy for liver injury, packing is initially attempted and if it controls bleeding and the patient is still unstable, a damage control approach is taken*.* However, if persistent bleeding is encountered, a timely decision must be made to proceed with hepatic resection. As demonstrated in the case, a right hepatectomy was the most effective way of controlling bleeding. The initial management, albeit suboptimal, age and minimal transfusion requirements may have led the patient to endure such major hepatic resection. The resuscitative efforts in the ICU perioperatively and the assistance of the anesthesiologists were also crucial in avoiding the triad of massive blood loss, hypothermia and acidosis leading to irreversible coagulopathy.

In conclusion, an anatomic or non-anatomic hepatic resection is a viable and safe option for traumatic and complex liver injuries. Early decision by a dedicated team of surgeons coupled with the necessary support from ancillary services is essential for an optimal outcome in these cases.

## Provenance and peer review

Not commissioned, externally peer-reviewed.

## Ethical approval

Yes, ethics approval was acquired thru the University of the Philippines Manila Research Grants Administration Office with reference number RGAO-2021-1409.

## Sources of funding

None.

## Consent

Yes. Consent was obtained from the patient.

## Author contribution

SP – writing the paper, final editing.

EA – data collection, study design, manuscript editing.

AC – data collection, review of literature, writing the paper.

JM – data collection, review of literature, final draft.

OO – writing the paper, final draft.

## Registration of research studies

1. Name of the registry: N/A.

2. Unique Identifying number or registration ID: N/A.

3. Hyperlink to your specific registration (must be publicly accessible and will be checked): N/A.

## Guarantor

Siegfredo R. Paloyo, MD, MPH.

## Declaration of competing interest

None.
